# Antiviral Properties of Chemical Inhibitors of Cellular Anti-Apoptotic Bcl-2 Proteins

**DOI:** 10.3390/v9100271

**Published:** 2017-09-25

**Authors:** Daria Bulanova, Aleksandr Ianevski, Andrii Bugai, Yevhen Akimov, Suvi Kuivanen, Henrik Paavilainen, Laura Kakkola, Jatin Nandania, Laura Turunen, Tiina Ohman, Hanna Ala-Hongisto, Hanna M. Pesonen, Marika S. Kuisma, Anni Honkimaa, Emma L. Walton, Valentyn Oksenych, Martina B. Lorey, Dmitry Guschin, Jungmin Shim, Jinhee Kim, Thoa T. Than, So Young Chang, Veijo Hukkanen, Evgeny Kulesskiy, Varpu S. Marjomaki, Ilkka Julkunen, Tuula A. Nyman, Sampsa Matikainen, Jani S. Saarela, Famara Sane, Didier Hober, Gülsah Gabriel, Jef K. De Brabander, Miika Martikainen, Marc P. Windisch, Ji-Young Min, Roberto Bruzzone, Tero Aittokallio, Markus Vähä-Koskela, Olli Vapalahti, Arto Pulk, Vidya Velagapudi, Denis E. Kainov

**Affiliations:** 1 Institute for Molecular Medicine Finland, FIMM, University of Helsinki, Helsinki 00290, Finland; daria.bulanova@helsinki.fi (D.B.); aleksandr.ianevski@helsinki.fi (A.I.); yevhen.akimov@helsinki.fi (Y.A.); jatin.nandania@helsinki.fi (J.N.); laura.turunen@helsinki.fi (L.T.); evgeny.kulesskiy@helsinki.fi (E.K.); jani.saarela@helsinki.fi (J.S.S.); tero.aittokallio@helsinki.fi (T.A.); markus.vaha-koskela@helsinki.fi (M.V.-K.); vidya.velagapudi@helsinki.fi (V.V.); 2 Department of Biochemistry and Developmental Biology, University of Helsinki, Helsinki 00290, Finland; andrii.bugai@helsinki.fi; 3 Department of Virology, University of Helsinki, Helsinki 00290, Finland; suvi.kuivanen@helsinki.fi; 4 Department of Virology, University of Turku, Turku 20520, Finland; hojpaa@utu.fi (H.P.); laura.kakkola@utu.fi (L.K.); veijo.hukkanen@utu.fi (V.H.); ilkka.julkunen@utu.fi (I.J.); 5 Institute of Biotechnology, University of Helsinki, Helsinki 00014, Finland; tiina.ohman@helsinki.fi (T.O.); t.a.nyman@medisin.uio.no (T.A.N.); 6 Biomedicum Functional Genomics Unit (FuGU), Helsinki, Helsinki 00290, Finland; hanna.ala-hongisto@helsinki.fi (H.A.-H.); hanna.m.pesonen@helsinki.fi (H.M.P.); marika.kuisma@helsinki.fi (M.S.K.); 7 Department of Virology, University of Tampere, Tampere 33520, Finland; Anni.Honkimaa@uta.fi; 8 Department of Clinical and Molecular Medicine, Norwegian University of Science and Technology, Trondheim 7028, Norway; emma.l.walton@ntnu.no (E.L.W.); valentyn.oksenych@ntnu.no (V.O.); 9 University of Helsinki and Helsinki University Hospital, Rheumatology, Helsinki 00290, Finland; martina.lorey@helsinki.fi (M.B.L.); sampsa.matikainen@helsinki.fi (S.M.); 10 Institut Pasteur Korea, Gyeonggi-do 13488, Korea; albanec2@gmail.com (D.G.); jungmin.shim@ip-korea.org (J.S.); jinhee.kim@ip-korea.org (J.K.); thoa.than@ip-korea.org (T.T.T.); soyoung.chang@ip-korea.org (S.Y.C.); marc.windisch@ip-korea.org (M.P.W.); jiyoung.min@ip-korea.org (J.-Y.M.); bruzzone@hkucc.hku.hk (R.B.); 11 Department of Biological and Environmental Science/Nanoscience center, University of Jyväskylä, Jyväskylä 40500, Finland; varpu.s.marjomaki@jyu.fi; 12 Department of Immunology, University of Oslo, Oslo 0424, Norway; 13 University of Lille, CHU Lille laboratoire de Virologie, EA3610, F-59037 Lille, France; famara.sane@chru-lille.fr (F.S.); didier.hober@chru-lille.fr (D.H.); 14 Heinrich Pette Institute, Leibniz Institute for Experimental Virology, Hamburg 20251, Germany; guelsah.gabriel@hpi.uni-hamburg.de; 15 Department of Biochemistry, University of Texas Southwestern Medical Center, Dallas, TX 75390-9038, USA; jef.debrabander@utsouthwestern.edu; 16 Department of Immunology, Genetics and Pathology, Science for Life Laboratory, Uppsala University, Uppsala 75237, Sweden; miika.martikainen@igp.uu.se; 17 HKU-Pasteur Research Pole, School of Public Health, University of Hong Kong, Hong Kong, China; 18 Department of Cell Biology and Infection, Institut Pasteur, Paris 75015, France; 19 Department of Virology and Immunology, University of Helsinki and Helsinki University Hospital, Helsinki 00014, Finland; olli.vapalahti@helsinki.fi; 20 Department of Veterinary Biosciences, University of Helsinki, Helsinki 00014, Finland; 21 Institute of Technology, University of Tartu, Tartu 50090, Estonia; arto.pulk@ut.ee

**Keywords:** apoptosis, antiviral agent, innate immunity, host response

## Abstract

Viral diseases remain serious threats to public health because of the shortage of effective means of control. To combat the surge of viral diseases, new treatments are urgently needed. Here we show that small-molecules, which inhibit cellular anti-apoptotic Bcl-2 proteins (Bcl-2i), induced the premature death of cells infected with different RNA or DNA viruses, whereas, at the same concentrations, no toxicity was observed in mock-infected cells. Moreover, these compounds limited viral replication and spread. Surprisingly, Bcl-2i also induced the premature apoptosis of cells transfected with viral RNA or plasmid DNA but not of mock-transfected cells. These results suggest that Bcl-2i sensitizes cells containing foreign RNA or DNA to apoptosis. A comparison of the toxicity, antiviral activity, and side effects of six Bcl-2i allowed us to select A-1155463 as an antiviral lead candidate. Thus, our results pave the way for the further development of Bcl-2i for the prevention and treatment of viral diseases.

## 1. Introduction

Globalization, environmental changes, population growth, and urbanization make viral diseases (VDs) one of the major causes of morbidity and mortality in the world [1,2]. In particular, emerging and re-emerging VDs pose a constant threat to public health [3]. To date, 90 antiviral drugs have been approved to treat human immunodeficiency virus (HIV) 1 and 2, hepatitis B and C viruses (HBV and HCV), cytomegalovirus (CMV), herpes simplex virus (HSV) 1 and 2, human papilloma virus (HPV), varicella zoster virus (VZV), vaccinia virus (VACV), Epstein-Barr virus (EBV), respiratory syncytial virus (RSV), or influenza A and B virus (IAV and FLUBV) infections but not other important VDs [4]. Only aciclovir (HSV-1/-2, VZV), famiciclovir (HSV-1/-2, VZV), valaciclovir (HSV-1/-2, VZV, EBV, CMV), vidarabine (HSV-1/-2, VZV), brivudine (HSV-1, VZV), foscarnet (HSV-1/-2, CMV), lamivudine (HBV, HIV-1/-2), peginterferon α-2a (HBV, HCV), peginterferon α-2b (HBV, HCV), ribavirine (RSV, HCV), tenofovir disoproxil (HBV, HIV-1/-2), and trifluridine (HSV-1/-2, VACV) can target more than one VD [4]. Considering this, there is an urgent need for the development of novel antivirals, including drugs against emerging and re-emerging VDs.

Apoptosis is a cellular antiviral process that can be exploited in development of such drugs [5,6]. During this process, pattern recognition receptors (PRRs) recognize the invading viruses and signal to Bcl-2 proteins [7]. The anti-apoptotic (Bcl-2, Bcl-xL, and Bcl-w) and pro-apoptotic (Bax, Bak, and Bad) Bcl-2 proteins associate or dissociate with each other using Bcl-2-homology 3 (BH3) domains to start a cascade of events, which leads to mitochondria membrane permeabilization (MoMP), cytochrome c release, and eventually cell death [8,9,10,11].

Anti-apoptotic Bcl-2 proteins may represent cellular targets for novel antivirals [12,13,14]. Moreover, commercially available Bcl-2 inhibitors (Bcl-2i) could be tested as antivirals. In particular, the antiviral properties of two structurally distinct classes of anticancer agents could be investigated. The first class includes ABT-737 and its derivatives ABT-263 and ABT-199, whereas the second class includes WEHI-539 and its derivatives, A-1331852 and A-1155463 [15,16,17,18,19,20,21]. Interestingly, ABT-263 is currently in clinical trials, and ABT-199 is approved to treat multiple lymphoid malignancies. However, only A-1155463 causes reversible thrombocytopenia in animals [22,23].

Here, we showed that ABT-263 limited the replication of IAV, FLUBV, Middle East respiratory syndrome coronavirus (MERS-CoV), Zika virus (ZIKV), HBV, HSV-1 and -2, and echovirus 1 and 6 (EV1 and EV6) by inducing the premature death of virus-infected cells at concentrations not toxic for non-infected cells. We further showed that ABT-263 induced apoptosis in cells transfected with viral RNA or plasmid DNA but not in mock-transfected cells, suggesting that this compound accelerated apoptosis in response to foreign nucleic acids. We also demonstrated that ABT-737, ABT-199, WEHI-539, A-1331852, and A-1155463 can specifically induce the premature death of IAV-infected cells, but only ABT-737, ABT-263, A-1331852, and A-1155463 limited viral replication. A comparison of the toxicity, antiviral activity, and side effects of these Bc2i allowed us to select A-1155463 as an antiviral lead candidate. Thus, we discovered a potential solution for the prevention and treatment of a broad-spectrum of VDs.

## 2. Materials and Methods

### 2.1. Reagents

ABT-199, ABT-263, ABT-737, WEHI-539, A-1331852, A-1155463, obatoclax, and gemcitabine were purchased from Selleck Chemicals (Houston, TX, USA), Saliphenylhalamide (SaliPhe) was synthesized as described previously [24]. 10 mM solutions of the compounds were prepared in 100% dimethyl sulfoxide (Sigma-Aldrich, St. Louis, MO, USA) and stored at −80 °C. Lyophilized lipopolysaccharide (LPS, 10 mg/mL) was purchased from Sigma-Aldrich (St. Louis, MO, USA). Plasmid pEGFP was from Clontech (Mountain View, CA, USA) (cat #: HLP309). Hoechst 33342 (20 mM solution; cat #: 62249) and ATP (10 mM solution; cat #: PV3227) were from Thermo Fisher Scientific (Waltham, MA, USA). Genomic RNA was isolated from influenza A/WSN/1933 strain as described previously [11].

### 2.2. Viruses

Human influenza A/Udorn/307/1972 (H3N2) and B/Shandong/7/97 viruses were grown in embryonated hen eggs as described previously [25,26]. EV1 and EV6 strains were propagated in a monolayer of african green monkey kidney (Vero) and adenocarcinomic human alveolar basal epithelial A549 cells, respectively, as described earlier [27,28]. Vero cells were used to prepare HSV-1 and -2 stocks as described previously [29]. ZIKV FB-GWUH-2016 strain was cultured in Vero E6 cells as described earlier [30]. Semliki Forest virus (SFV) expressing VA7-mCherry was generated by the in vitro transcription of pSP6-SFV4 cDNA and the electroporation of mRNA in baby hamster kidney (BHK) 21 cells as previously described [31]. HBV particles were prepared from the culture supernatant of HepAD38 cells as described previously [32]. MERS-CoV was propagated in a monolayer of Vero cells for three days at 37 °C as previously described [33]. The virus stocks were stored at −80 °C. All the experiments with viruses were performed in compliance with the guidelines of the national authorities using appropriate Biosafety laboratories under appropriate ethical and safety approvals.

### 2.3. Cells

All cells were propagated at 37 °C in 5% CO_2_. Human retinal pigment epithelial (RPE) cells were grown in Dulbecco's modified Eagle's medium (DMEM) F12 supplemented with 50 U/mL penicillin-streptomycin (PenStrep), 2mM L-glutamine, 10% fetal bovine serum (FBS), and 0.25% sodium bicarbonate (Sigma-Aldrich). Virus growth medium (VGM) for RPE contained 0.2% BSA, 2 mM l-glutamine, 0.348% NaHCO_3_, and 1 μg/mL l-1-tosylamido-2-phenylethyl chloromethyl ketone-trypsin (TPCK)-trypsin (Sigma-Aldrich) in DMEM-F12. Human A549 cells were grown in DMEM medium, supplemented with 50 U/mL PenStrep, 2 mM l-glutamine, and 10% FBS. VGM for A549 contained 0.2% BSA, 2 mM l-glutamine, and 1 μg/mL 1-tosylamido-2-phenylethyl chloromethyl ketone-trypsin (TPCK)-trypsin (Sigma-Aldrich) in DMEM. Pa02C were grown in regular DMEM supplemented with 10% FBS. HepG2-NTCP2 cells were maintained in DMEM supplemented with 10% FBS, 2 mM l-glutamine, 50 U/mL penicillin, and 50 µg/mL streptomycin as previously described [32]. Vero cells were grown in high glucose DMEM supplemented with 10% heat-inactivated FBS and 1× Antibiotic-Antimycotic solution (Gibco/Life Technologies, Carlsbad, CA, USA).

### 2.4. Compound Toxicity and Efficacy Assays

Approximately 4 × 10^4^ cells were seeded in each well of a 96-well plate. The cells were grown for 24 h in appropriate cell growth medium. The media was replaced with VGM containing CellTox Green Express cytotoxicity reagent (CTxG, 1:2000 dilution in the assay well, Promega, Madison, WI, USA). The compounds were added to the cells in three-fold dilutions at seven different concentrations starting from 10 μM. No compounds were added to the control wells. The cells were mock- or virus- infected. The multiplicity of infection (moi) was 0.1 to 3 depending on the virus strain. When Bcl-2i induced a cytopathic effect in virus-infected cells (typically on 12–24 h), the cells were imaged using a Cytation 5 Imaging reader (BioTek Instruments Inc., Winooski, VT, USA) in a bright-field or fluorescent mode (485–500 nm_Ex_/520–530 nm_Em_) and the fluorescence was measured with a PHERAstar FS plate reader (BMG Labtech, Ortenberg, Germany). The media was removed from the cells. CellTiter-Glo viability (CTG, Promega, Madison, WI, USA) reagent was added (30 µL per well). The luminescence was measured with a PHERAstar FS plate reader. The raw data was normalized against calibration (standard) curves. The curves were generated using purified human genomic DNA or ATP (Sigma Aldrich). Each of the curves was fitted using logistic regression analysis, with *R*^2^_PRESS_ = 0.997 and *R*^2^_PRESS_ = 0.995 for the dsDNA and ATP calibration curves, where *R*^2^_PRESS_ is a predicted coefficient of determination *R*-squared calculated from the predicted residual error sum of squares (PRESS) statistic [34]. This allowed accurate estimation of unknown concentrations by finding intersects with known fluorescence and luminescence measurements on the calibration curve.

### 2.5. Scoring Drug Response Profiles

The dose-response curves were fitted with four-parameter logistic functions (Equation (1)), where $A_{min\;}$and $A_{max\;}$are the upper and lower asymptotes (minimal and maximal drug effects), m is the dose that produces the half-maximal effect, and λ is the steepness (slope) of the curve.

(1) $$f\left(x\right)=\;A_{min}+\;\frac{A_{max}-\;A_{min}}{1+{\left(\frac{x}{m}\right)}^{\lambda }}$$

We used a composite trapezoidal rule to estimate the area under the dose-response curve (AUC), which is a commonly used metric for the quantification of drug responses through multiple dose-levels [35,36,37]. This allowed us to calculate differential AUC (dAUC) between two dose-response curves (for example, plus minus virus).

The half maximal cytotoxic concentration (CC_50_), the half maximal effective concentration (EC_50_) for each compound was determined as described previously [38]. The relative effectiveness of the drug was defined as the therapeutic or selectivity index (SI = CC_50_/EC_50_).

### 2.6. Virus Titration

The cells were treated with 1 µM Bcl-2i or remained non-treated and infected with a virus. After 24 h, supernatants were collected, serially diluted in PBS, and added to Vero-E6, MDCK, or A549 cells. The media was changed, and the cells were overlaid with plaque assay media. The cells were fixed, and viral titers were calculated. The titers were expressed as plaque-forming units (PFU), 50% tissue culture infective dose (TCID50), or fluorescence-forming units (FFU)/mL. The EC_50_ values as well as the ratios between virus titers in non-treated and compound-treated cells at certain Bcl-2i concentrations were calculated.

IAV and FLUBV were titered using plaque assay on MDCK cells, as described earlier [25]. EV1 and EV6 titers were also determined by plaque assay on A549 cells [39]. HSV-1 and HSV-2 titers were determined by plaque titration in Vero cells in the presence of human immunoglobulin G (20 µg/mL) as described earlier [29]. SFV4 was titered by plaque assay on BHK-21 cells, as previously described [31].

### 2.7. Transfections of RPE Cells with vRNA or Plasmid DNA

RPE cells were cultured to 80% confluence in 96 well plates and transfected with 160 ng viral genomic RNA using Lipofectamine RNAiMAX (Thermo Fisher Scientific, Waltham, MA, USA) or with 30, 100, or 300 ng of plasmid DNA (pEGFP) using Lipofectamine 3000 (Thermo Fisher Scientific, Waltham, MA, USA).

### 2.8. Live Microscopy of Semliki Forest Virus Infection During Bcl-2-Inhibition

One thousand Pa02C cells were seeded per well in 384-well plates in duplicate in the presence of 1:2000 dilution of CellToxGreen reagent. Cells were infected with 10 PFUs per well of SFV vector VA7-mCherry [31]. Plates were placed in an Incucyte Zoom live microscope (Essen Bioscience, Ann Arbor, MI, USA) and set to image every 30 min. Movies were assembled using all frames, while still images show the cells every 2 h.

### 2.9. Dynamic BH3 Peptide Profiling

Dynamic BH3 profiling was performed as described previously [40]. Briefly, 4 × 10^4^ RPE cells were plated per well in 96-well plates (Corning, Corning, NY, USA). After 16 h, the cells were infected with IAV or transfected with pEGFP plasmid using Lipofectamine 3000 following the manufacturer’s instructions. Three hours post infection and one hour post transfection, the medium was replaced with 100 μL of Derived from Trehalose Experimental Buffer (DTEB). The DTEB contained 135 mM trehalose, 50 mM KCl, 20 μM EDTA, 20 μM EGTA, 0.1% BSA, 5 mM succinate, 10 mM HEPES-KOH, 0.005% digitonin, 10 μg/mL Oligomycin A, 5 μM β-mercaptoethanol, 1 μM JC-1 fluorescent probe (all from Sigma), and BH3-domain peptides BIM (1 μM), BID (5 μM), PUMA (5 μM), NOXAA (5 μM), BAD (10 μM), HRK (5 μM; KJ Ross-Peterssen, ApS, Copenhagen, Denmark), or DMSO (Sigma-Aldrich). The fluorescent signal was measured at λ = 593 nm for 150 min with 10 min intervals using a Cytation 5 Imaging reader (BioTek Instruments Inc., Winooski, VT, USA). The kinetic curves were plotted and analyzed using GraphPad Prism7 (GraphPad Software Inc., La Jolla, CA, USA). Mitochondrial depolarization and delta apoptotic priming were calculated as described [40].

### 2.10. Metabolomics

Metabolomics analysis was performed as described previously [41]. Briefly, 10 μL of labeled internal standard mixture was added to 100 μL of the sample (cell culture media), and 0.4 mL of solvent (99% ACN and 1% FA) was added to each sample. The samples were filtered. Sample analysis was performed on an Acquity UPLC-MS/MS system (Waters Corporation, Milford, MA, USA). The detection system, a Xevo TQ-S tandem triple quadrupole mass spectrometer (Waters, Milford, MA, USA), was operated in both positive and negative polarities with a polarity switching time of 20 msec. Electro spray ionization was chosen as the ionization mode with a capillary voltage at 0.6 kV in both polarities. The source temperature and desolvation temperature of 120 °C and 650 °C, respectively, were maintained constantly throughout the experiment. The declustering potential and collision energy were optimized for each compound. The Multiple Reaction Monitoring (MRM) acquisition mode was selected for the quantification of the metabolites with an individual span time of 0.1 s given in their individual MRM channels. The dwell time was calculated automatically by the software based on the region of the retention time window, the number of MRM functions, and also depending on the number of data points required to form the peak. MassLynx software (version 4.1, Waters Corporation, Milford, MA, USA) was used for data acquisition, data handling, and instrument control. Data processing was done using TargetLynx software (Waters Corporation, Milford, MA, USA), and the metabolites were quantified by calculating the curve area ratio using labeled internal standards and external calibration curves. The metabolomics data was log2 transformed for linear modeling and empirical-Bayes-moderated *t*-tests using the R/Bioconductor software package *limma* [42]. To analyse the differences in metabolites levels, a linear model was fit to each metabolite. The Benjamini-Hochberg method was used to correct for multiple testing. The significant metabolites were determined at a Benjamini-Hochberg false discovery rate (FDR) controlled at 10%. The heatmap was generated using the pheatmap package based on log transformed profiling data. MetaboAnalyst (version 3.0, McGill University, Ste. Ann de Bellevue, QC, Canada) was used to identify the metabolic pathways associated with virus infection or affected by Bcl-2i treatment [43].

### 2.11. Immuno-Precipitation and Mass-Spectrometry

The Bcl-xL-, Bcl-2-, or Mcl-1-associated factors were immuno-precipitated from IAV-infected and non-infected RPE cells using rabbit anti-Bcl-xL, Bcl-2, or Mcl-1 antibodies (1:200; Cell Signalling Technology, Danvers, MA, USA), separated with sodium dodecyl sulfate polyacrylamide gel electrophoresis (SDS-PAGE) and visualized by Coomassie staining. The entire lanes or specific protein bands were cut. The proteins were in-gel digested with trypsin. The resulting peptides were analyzed using liquid chromatography–tandem mass spectrometry, as described previously [11,44]. The mass spectrometry data were searched using in-house Mascot and the ProteinPilot interface against the SwissProt database. Only statistically significant data (*p* < 0.05) were selected.

## 3. Results

Our dynamic BH3 peptide profiling revealed that Bad, Bim, Bid, Puma, and Noxa enhanced MoMP in IAV- but not in mock-infected human non-malignant RPE cells, which represent natural targets for IAV infection (Figure S1) [45,46,47,48,49,50]. A co-immunoprecipitation experiment using antibodies against pro-survival Bcl-xL, Bcl-2, or Mcl-1 followed by mass spectrometry showed that several cellular proteins, including Bad, Bax, Bak, UACA, PAWR, FLII, Trim21, IMMT, 14-3-3, EFHD2, DHX9, DDX3, NLRP3, and LRRFIP2, as well as viral factors M1, NS1, HA, and NP were present in the complexes (Figure S2). Thus, these experiments demonstrated that pro-apoptotic Bcl-2 proteins (Bad, Bax, Bak), PRRs (DHX9, DDX3, LRRFIP2), and other factors can be involved in the programmed death of IAV-infected cells.

**Figure 1:**
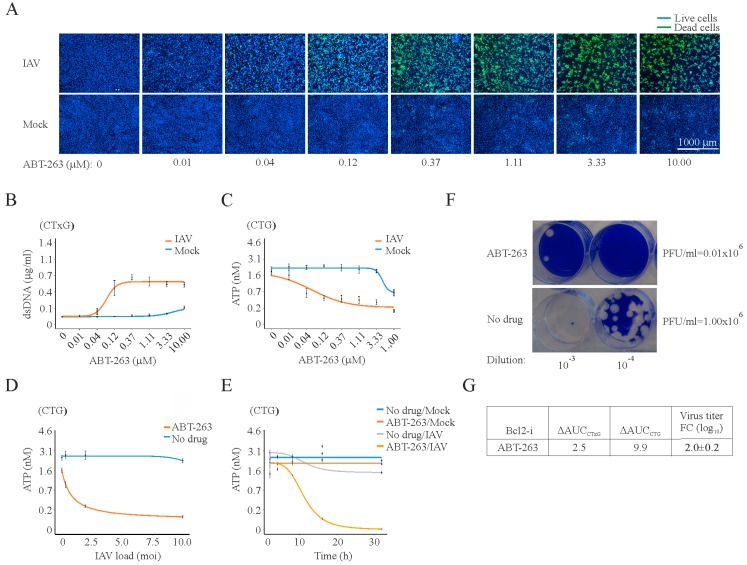
At 24 h post infection, ABT-263 kills influenza A (IAV)-infected but not mock-infected RPE cells and lowers the production of infectious viral particles. (**A**) Fluorescent microscopy images showing that increasing concentrations of ABT-263 kill IAV-infected (moi 3) but not mock-infected retinal pigment epithelium (RPE) cells at 24 h. Asymmetric cyanine dye stains the dsDNA of dead cells. Hoechst stains DNA in living cells; (**B**) quantification of dsDNA in dead cells using CellToxGreen cytotoxicity (CTxG) assay. Mean ± standard deviation (SD), *n* = 3; (**C**) quantification of intracellular ATP in living cells using CellTiter-Glo luminescent cell viability (CTG) assay. Mean ± standard deviation (SD), *n* = 3; (**D**) RPE cells were non- or ABT-263-treated (0.4 μM) and infected with IAV at moi 0.08, 0.4, 2, and 10. Cell viability was measured using a CTG assay 24 h after infection. Mean ± SD, *n* = 3; (**E**) RPE cells were non- or ABT-263-treated (0.4 μM) and mock- or IAV-infected (moi 3), and cell viability was measured using a CTG assay at the indicated time points. Mean ± SD, *n* = 3; (**F**) example of plaque assay measuring virus production in Bcl-2i- (3 µM) and DMSO-treated RPE cells at 24 hpi; (**G**) table summarizing the differential effect of ABT-263 on the viability of virus- and mock-infected cells, expressed as ΔAUC_CxTG_ and ΔAUC_CTG_. It also shows the effect of ABT-263 on virus production in drug- (3 µM) and DMSO-treated RPE cells, which is expressed in log_10_ fold change (FC). Mean ± SD, *n* = 3.

It was shown that ABT-263 targets Bcl-xL and Bcl-2 and alters their interaction with pro-apoptotic Bax, Bad, and Bak [19,20]. We tested the effect of ABT-263 on the viability of RPE cells infected with IAV or mock by carrying out dose response studies. As readouts, we used fluorescent microscopy, which visualizes dead (green) and living (blue) cells. Fluorescent microscopy revealed that ABT-263 induced the premature death of IAV-infected cells at concentrations not toxic for non-infected cells (Figure 1A).

We validated the results with the CTxG and CTG assays. The CTxG assay uses fluorescent asymmetric cyanine dye that stains the DNA of dead cells, whereas CTG assay quantifies ATP, an indicator of metabolically active living cells. We calculated the values of the differential area under the dose-response curves between the mock- and IAV-infected cells for both assays (ΔAUC_CTG_ and ΔAUC_CTG_; Figure 1B,C,E). High values indicate that ABT-263 was an effective trigger of the premature death of infected cells. In infected cells, the effect was observed already at 12 h post infection, whereas no toxicity was detected for at least 36 h at 0.4 µM ABT-263 in the mock-infected cells (Figure 1E). Moreover, the effect was dependent not only on the dose of ABT-263 but also on the viral load (Figure 1D).

In addition, we evaluated the effect of ABT-263 on IAV replication by tittering viruses produced in cells treated and non-treated with ABT-263. ABT-263 treatment lowered IAV production in RPE cells, as indicated by the fold change between virus titers (Figure 1D,E). We obtained similar results in A549 cells (Figure S3). Furthermore, ABT-263 activated caspase 3 in IAV-infected but not in non-treated or ABT-263-treated mock-infected RPE cells at 12 h post infection (Figure S4). These results collectively suggest that ABT-263 induced premature apoptosis in IAV-infected cells and lowered IAV production.

**Figure 2:**
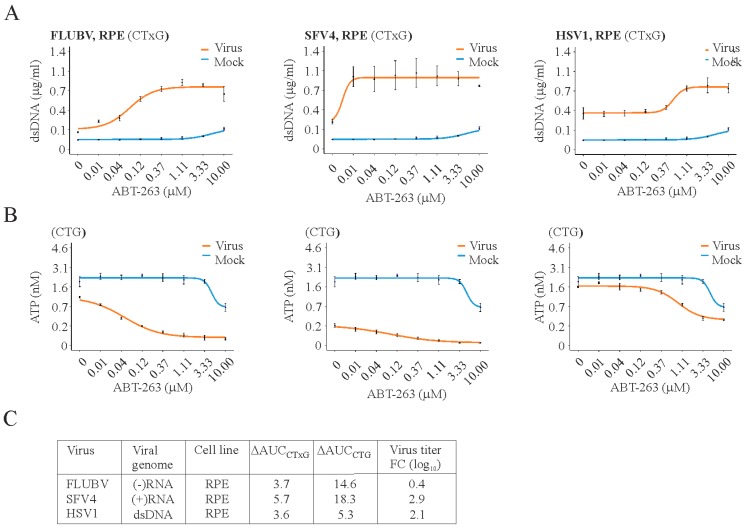
ABT-263 induces the premature death of cells infected with different viruses. (**A**) CTxG and (**B**) CTG plots showing the ABT-263 induces the premature death of RPE cells infected with FLUBV, SFV4, or HSV-1 viruses (moi 1) but not those infected with mock. Mean ± SD, *n* = 3; (**C**) table summarizing the differential effect of Bcl-2i on the viability of virus- and mock-infected cells, expressed as ΔAUC_CxTG_ and ΔAUC_CTG_. It also shows the effect of ABT-263 on virus production in drug- (3 µM) and DMSO-treated RPE cells, which is expressed in log_10_ fold change (FC).

Importantly, ABT-263 induced the premature death of RPE cells infected with FLUBV, SFV, or HSV-1 and lowered virus production, all at concentrations that are not toxic to non-infected cells (Figure 2). We obtained similar results with ZIKV, EV1, EV6, MERS-CoV, HBV, and HSV-2 viruses using different cell lines (Figure S5). Supplementary movies 1 and 2 further reaffirm that the treatment of pancreatic cancer cells infected with SFV with ABT-263 at low moi attenuated the replication and spread of the virus in these cells. These results suggest that Bcl-2i might be used as broad-spectrum antivirals.

**Figure 3:**
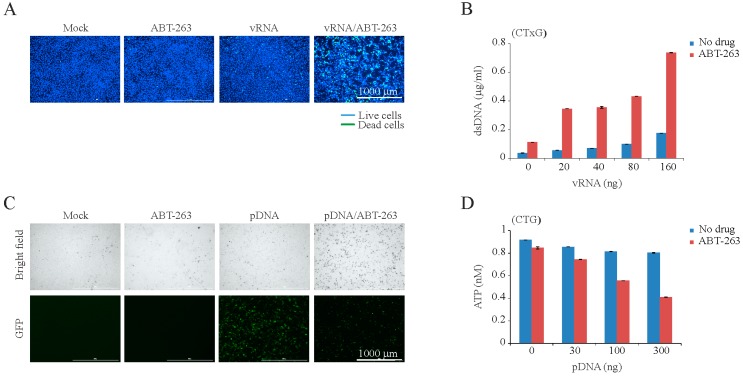
ABT-263 induces the premature death of cells transfected with IAV genomic RNA (vRNA) or plasmid DNA (pDNA). (**A**) Fluorescent microscopy images showing that ABT-263 kills vRNA-transfected (160 ng) but not mock-transfected RPE cells at 8 h post transfection. Asymmetric cyanine dye stains the dsDNA of dead cells. Hoechst stains DNA in living cells; (**B**) CTxG plot showing that ABT-263 (3 µM) induces that premature death of RPE cells transfected with increasing concentrations of vRNA. Mean ± SD, *n* = 3; (**C**) Fluorescent and bright field microscopy of RPE cells showing that ABT-263 kills eGFP-expressing plasmid transfected (300 ng) but not mock-transfected RPE cells at 6 h post transfection; (**D**) CTG graph showing that the viability of ABT-263-treated (3 µM) cells decreases with increasing concentrations of transfected plasmid DNA. Mean ± SD, *n* = 3.

We hypothesized that viral or any other foreign RNA or DNA could trigger ABT-263-sensitized apoptosis. To test this, we transfected RPE cells with IAV RNA (vRNA) or plasmid DNA (pDNA) and treated transfected cells with ABT-263. Viral RNA- or pDNA, but not mock-transfected cells, rapidly died after ABT-263 treatment (Figure 3 and Figure S6 A,B). It is important to note that cell death was dependent on the amount of transfected vRNA and pDNA, as well as on concentration of ABT-263. Moreover, BH3 peptides of Puma, Bad, and Bid induced MoMP in pDNA-transfected ABT-263-treated cells as early as 3 h post transfection, in contrast to cells treated with ABT-263 or cells transfected with pDNA (Figure S6 F). As expected, ABT-263 did not accelerate the death of RPE or A549 cells treated with bacterial lipopolysaccharides (LPS), which are recognized by different PRRs to those that detect intracellular foreign RNA or DNA (Figure S6C–E).

In the next experiment, we pre-treated RPE or A549 cells with compounds that inhibit the endocytic uptake (obatoclax or SaliPhe) or the transcription and replication of IAV (JNJ-872 or gemcitabin) [46,51] and infected them with IAV. ABT-263 did not show any effect on these cells (Figure S7). These results confirmed that intracellular foreign RNA/DNA or its replication intermediates triggered apoptosis, and Bcl-2i accelerate this process.

**Figure 4:**
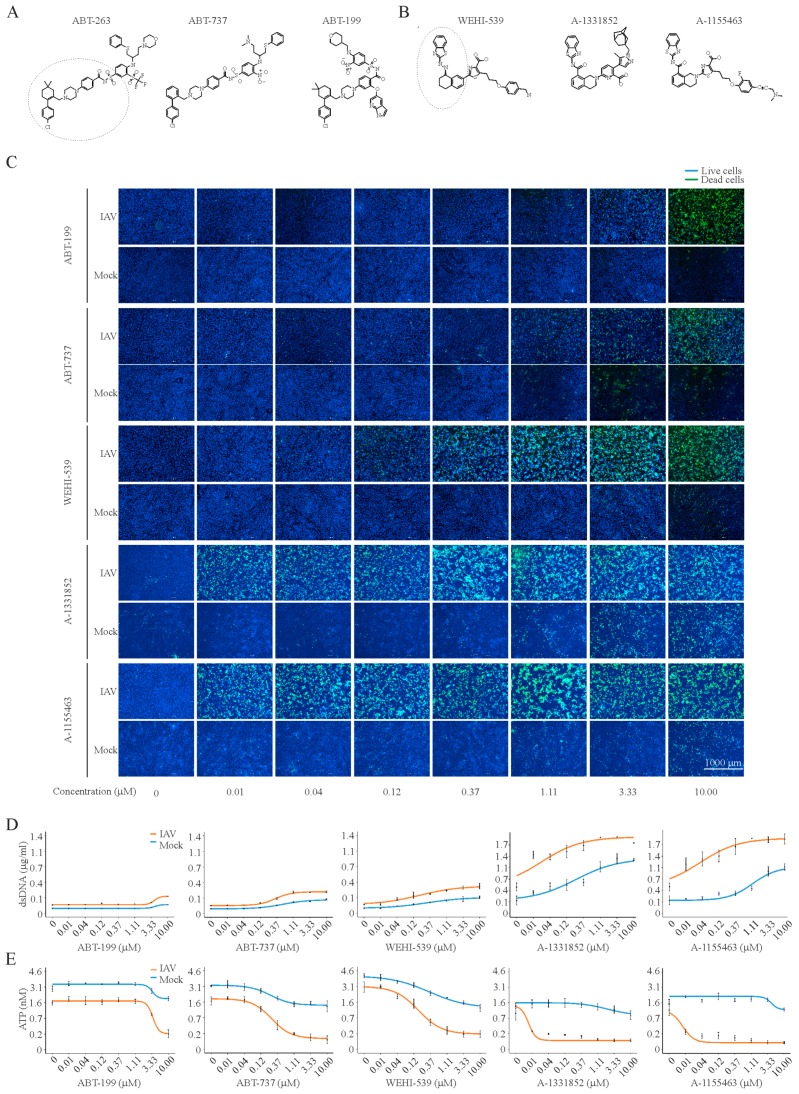
Anti-IAV activities of ABT-263 analogues. (**A**,**B**) Structures of ABT-263, ABT-737, and ABT-199, as well as WEHI-539, A-1331852, and A-1155463, showing that these molecules share similar elements; (**C**) fluorescent microscopy images showing that increasing concentrations of Bcl-2i kill IAV-infected (moi 3) but not mock-infected RPE cells at 24 hpi. Asymmetric cyanine dye stains the dsDNA of dead cells. Hoechst stains DNA in living cells; (**D**) quantification of dsDNA in dead cells. Mean ± SD, *n* = 3; (**E**) quantification of intracellular ATP in living cells using CTG assay. Mean ± SD, *n* = 3.

**Figure 5:**
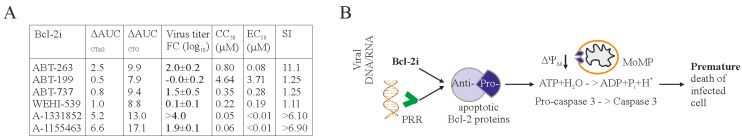
Bcl-2i facilitates apoptosis in virus infected cells. (**A**) Table showing the cytotoxic and antiviral activities of different Bcl-2 inhibitors; (**B**) schematic diagram showing how selective Bcl-2i induce the premature apoptosis of cells containing viral nucleic acids.

In order to provide further clues on the mechanisms of Bcl-2i-sensitized apoptosis, we investigated the effect of ABT-263 analogues (ABT-737, ABT-199, WEHI-539, A-1331852, and A-1155463) on the viability and death of IAV-infected and mock-infected RPE cells at 24 hpi (Figure 4). In addition, we evaluated the effect of these compounds on IAV replication by titering the viruses produced in cells treated with increasing compound concentrations. We calculated CC_50_, EC_50_, and SI values for each compound. We observed that ABT-263 was more selective than structurally similar ABT-737 and ABT-199, whereas A-1155463 was more selective than structurally similar A-1331852 and WEHI-539 (Figure 4 and Figure 5A). Importantly, the treatment of non-infected RPE cells with these compounds altered the levels of secreted adenine, adenosine, hypoxanthine, IMP, AMP, inosine, and xanthosine, which belong to the ATP metabolic pathway (Figure S8). Altogether, these results suggest that A-1155463 may represent an antiviral lead candidate.

## 4. Discussion

Generally, antivirals interfere with virus replication to prevent cell death. This can be achieved by the inhibition of critical steps of the virus replication cycle either by direct virus targeting or by targeting a host cell function. Here, we described an alternative approach, in which small molecules limited virus replication and spread by inducing the death of infected cells without affecting non-infected cells.

We showed that ABT-263 induced the premature apoptosis of IAV-infected cells at physiologically relevant concentrations. ABT-263 also facilitated apoptosis in cells infected with FLUBV, SFV, ZIKV, EV1, EV6, MERS-CoV, HBV, HSV-1, and HSV-2 or transfected with IAV vRNA or plasmid DNA. We concluded that foreign RNA/DNA triggered apoptosis in ABT-263-sensitized cells. It should be noted that other factors (such as IAV M2, PB1-F2, NS1, HA, and NP proteins, or HSV ICP27, ICP4, ICP10 PK, Us3, gD, and gJ) may further accelerate or decelerate apoptosis by disrupting or stabilizing the interactions of cellular BH3-domain proteins in infected cells [11,52,53,54,55].

Furthermore, we demonstrated that ABT-263-sensitized apoptosis was dependent on the load of viral nucleic acids. In particular, ABT-263 treatment induced apoptosis during the early stages of a viral infection if the viral load was high or during the late stages of a viral infection if the initial concentration of viral nucleic acid was low. In both cases, ABT-263 treatment could prematurely terminate viral replication. Mechanistically, PRRs recognized intracellular viral RNA/DNA and sent signals to anti-apoptotic Bcl-2 proteins. Bcl-2 proteins released their pro-apoptotic partners to initiate MoMP, ATP degradation and caspase-3 activation. This resulted in cell death. ABT-263 acted synergistically with viral RNA/DNA and, thereby, facilitated cell death (Figure 5B).

We also demonstrated that ABT-737, ABT-199, WEHI-539, A-1331852, and A-1155463 specifically induced the premature death of IAV-infected cells, but only ABT-737, ABT-263, A-1331852, and A-1155463 limited viral replication. Importantly, ABT-263 was more effective than structurally similar ABT-737 and ABT-199, whereas A-1155463 was more effective than structurally similar WEHI-539 and A-1331852. A-1155463, unlike ABT-263, causes reversible thrombocytopenia, which made it a suitable antiviral lead candidate [22,23].

The structures of A-1155463 and its analogues bound to Bcl-xL may provide further ideas for the development of this compound as an antiviral (Figure S9) [15,19,21,22,23]. The binding of A-1155463, A-1331852, and WEHI-539 to their targets induces major conformational change (7 Å backbone movement) in the Bcl-xL that disrupts the local (R103-D107) helical structure of the protein. This structural change is not seen with ABT-263, ABT-737, and ABT-199 binding as these compounds contain chlorophenyl moiety compared to A-115463, A-1331852, and WEHU-539 benzothiazole moiety. Moreover, A-1155463 has additional moiety, which perturbs the Bcl-xL structure and may affect its binding with its anti-apoptotic partners (such as Bad, Bax, and Bak) and, perhaps, other proteins (such as DHX9, LRRFIP2, PAWR, UACA, NS1, M1, NP, and HA). It is possible that, not only the high affinity of A-1155463 to Bcl-xL protein, but also its capacity to alter protein-protein interactions could be important for its pro-apoptotic antiviral effect.

Several critical issues should be also considered while developing A-1155463 or its derivatives as antivirals. While A-1155463 may possess broad-spectrum antiviral activity in vitro and against some other viruses in primary patient samples ex vivo [12,13,14], its antiviral efficacy should be evaluated in vivo. Also its side effects, including its immuno- and neuro-modulatory properties, should be studied in detail. In particular, A-1155463 or its derivatives may possess adverse effects in acute virus infection. The viral dose is likely to be high, infecting a large number of cells. Inducing apoptosis may result in extensive tissue damage. Thus, A-1155463 or its derivatives must be evaluated as prophylactic rather than as therapeutic agents against viruses that cause acute infections.

## 5. Conclusions

We can explore our knowledge in virus-host interaction to develop pharmacological interventions to control viral infections. In particular, our understanding of virus-mediated cell death highlighted the potential use of apoptosis-inducing molecules as antiviral drugs. Our study suggests that A-1155463 may be a lead pro-apoptotic compound with antiviral properties. The prophylactic use of A-1155463 and its derivatives might help to prevent severe viral diseases.

## Supplementary Materials

Supplementary materials can be found at http://www.mdpi.com/1999-4915/9/10/271/s1.
