# Supplementary material for: Antiviral Properties of Chemical Inhibitors of Cellular Anti-Apoptotic Bcl-2 Proteins
Source: Viruses. 2017 Sep 25;9(10):271. doi: 10.3390/v9100271 (PMC5691623; doi:10.3390/v9100271)
Supplement: Supplementary file 1 [file viruses-09-00271-s001.zip › Supplemental Figs.docx]

**Supplemental Material for Publication**

*“*Antiviral potential of anticancer Bcl-2 inhibitors*” by Bulanova et al.*

**
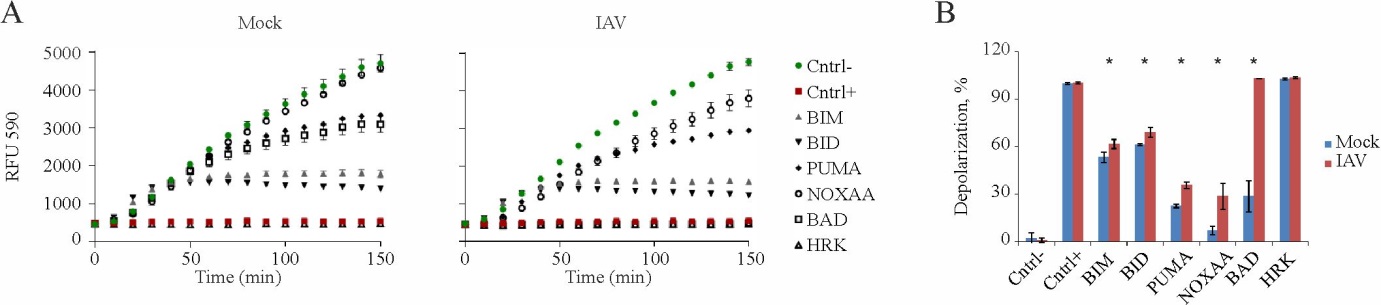
**

**Fig. S1**. Dynamic BH3-peptide profiling confirms that IAV infection mediates Bcl-2 dependent apoptosis. (A) RPE cells were infected with IAV or mock. After 3h, the medium was replaced with DTE buffer containing BH3-domain peptides, DMSO or FCCP and JC-1 fluorescent probe. Fluorescent signal at λ=593 nm was measured for 150 min with 10 min interval. Kinetic curves for mock- and IAV infected cells were plotted. (B) Mitochondrial membrane depolarization was calculated. The % depolarization was standardized to Cntrl+ (100%) and Cntr- (0%). Mean ± SD, n=3. Statistically significant (p<0.05, T-test) differences in mitochondrial membrane depolarization between virus- and mock-infected cells are indicated with asterisks.


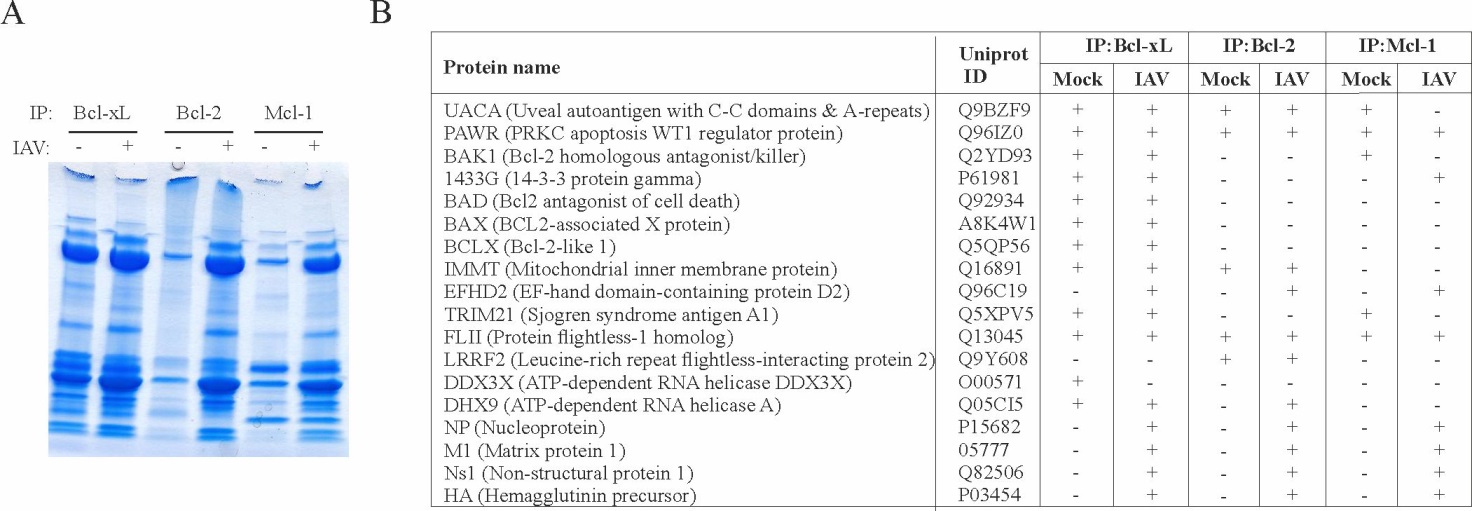


**Fig. S2**. Co-immuno-precipitation and mass spectrometry identified novel and confirmed known partners of Bcl-xL, Bcl-2 and Mcl-1, which may regulate apoptosis. RPE cells were mock- or IAV-infected (moi 3), Bcl-xL-, Bcl-2- and Mcl-1-interacting partners were co-immuno-precipitated using anti- Bcl-xL, Bcl-2 and Mcl-1 antibodies and analyzed using SDS-PAGE. The gel was stained with Coomassie. The entire lanes or specific bands of SDS-PAGE were cut, the proteins were in-gel digested and resulting peptides were analysed using LC-MS. The mass spectrometry data were searched against the SwissProt database. Selected proteins (excluding actin and myosin) are shown in the table.


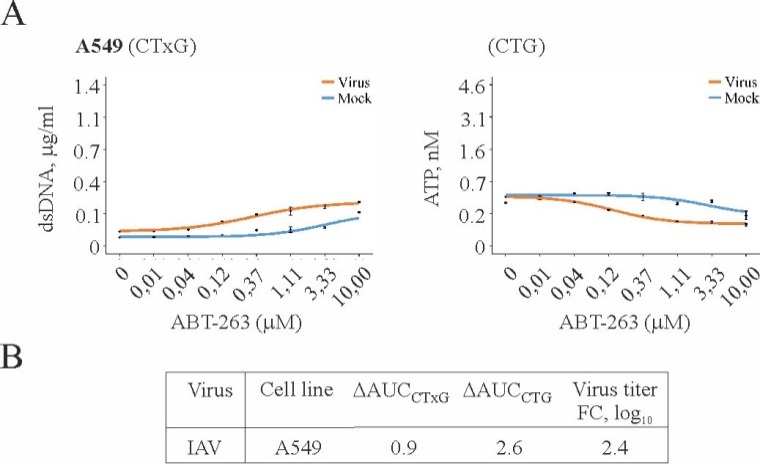


**Fig. S3**. At 24 h post infection, ABT-263 kills IAV-infected, but not mock-infected A549 cells, and lowers the production of infectious viral particles. A549 cells were treated with increasing concentration of ABT-263 or remained non-treated, and infected with mock or IAV (moi 3). After 24 h cell death and viability was measured by CTxG and CTG assays, respectively, and results were plotted.. Progeny viruses were tittered. Table shows the ΔAUC values and fold change in viral titers between ABT-263 treated (3 μM) and non-treated cells.

**
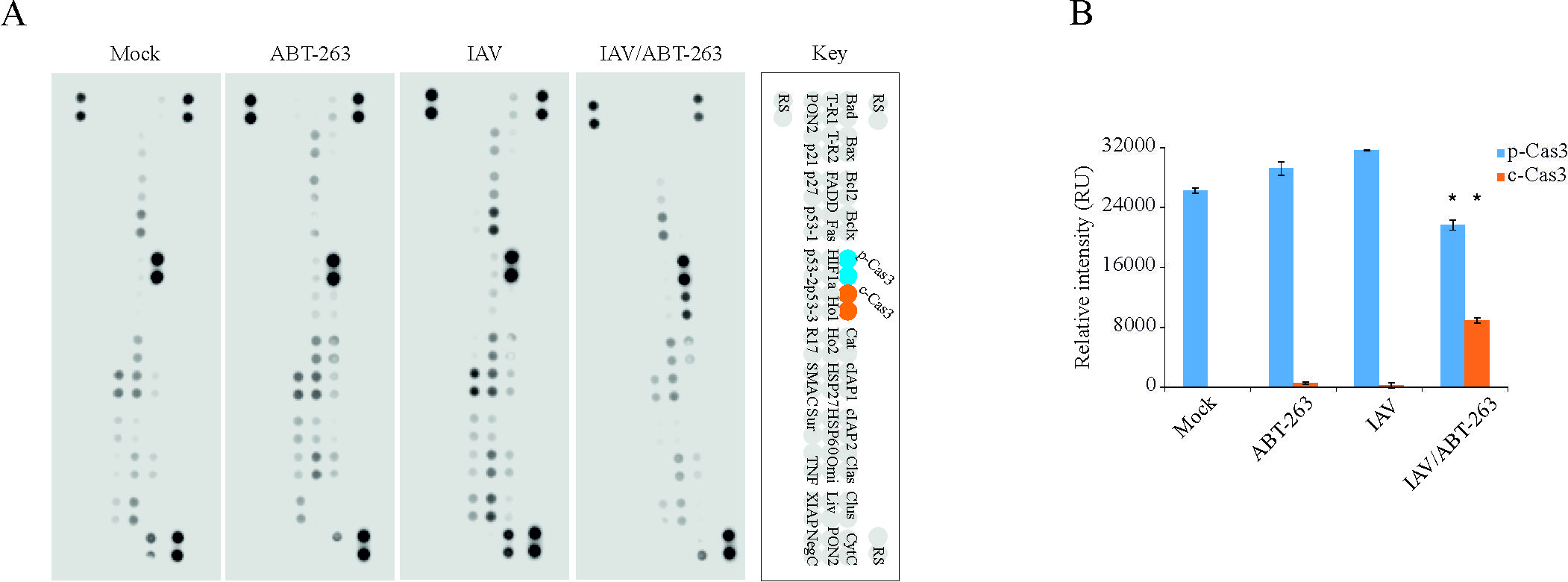
**

**Fig. S4**. Effect of IAV infection, ABT-263 treatment and their combination on caspase 3 activation. (A) RPE cells were treated with 1 μM ABT-263, or remained non-treated and infected with IAV (moi 1) or mock. After 12 h of infection 35 apoptosis related proteins were profiled using human apoptosis array kit. (B) Relative levels of pro-caspase 3 (p-Cas3) and cleaved Caspase 3 (c-Cas3) were calculated and plotted. Mean ± SD, n=3. Statistically significant (p<0.05, T-test) differences in pre-cleaved caspase-3 and cleaved caspase 3 levels between mock-infected, ABT-263-treated, IAV-infected and ABT-263-treated/IAV-infected cells are indicated with asterisks.


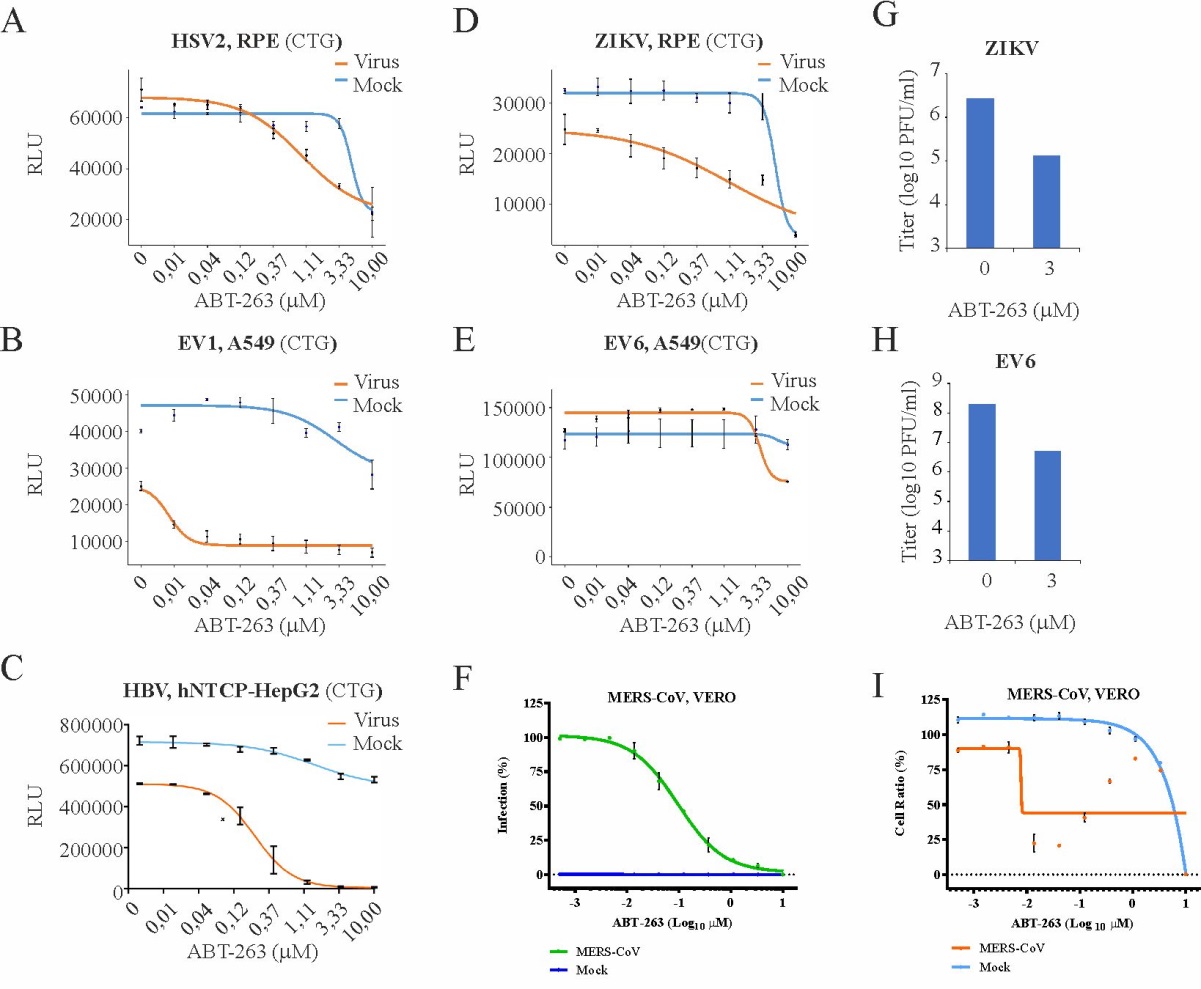


**Fig. S5**. Bcl-2i facilitate death of cells infected with different viruses. (A-E) Bcl-2i at non-cytotoxic concentrations facilitate death of HSV2- and ZIKV-infected RPE cells, EV1- and EV6-infected A549 cells, and HBV-infected hNTCP-HepG2 cells (moi=1-3). CTG plots showing that ABT-263 induces premature death of virus- but not mock-infected cells at 24 hpi. Mean ± SD, n=3. (F) ABT-263 blocks MERS-CoV replication in Vero cells at 24 hpi (moi 0.1). ABT-263 was added in dose-dependent manner to Vero cells. Cells were infected with MERS-CoV. Aftert 24 h cells were fixed with 4% paraformaldehyde. Percent of infection (green) was calculated by detecting MERS-CoV spike proteins in cells by immuno-fluorescent assay, and percent cell number (blue) was counted by Hoechst 33342 staining. Images were acquired by Perkin Elmer Operetta (20x) and analyzed by in-house developed Image Mining plug-in. Mean ± SD, n=3. (G-I) Graphs and plot showing the effect of ABT-263 on virus production in drug- and DMSO-treated RPE, A549 and Vero cells.


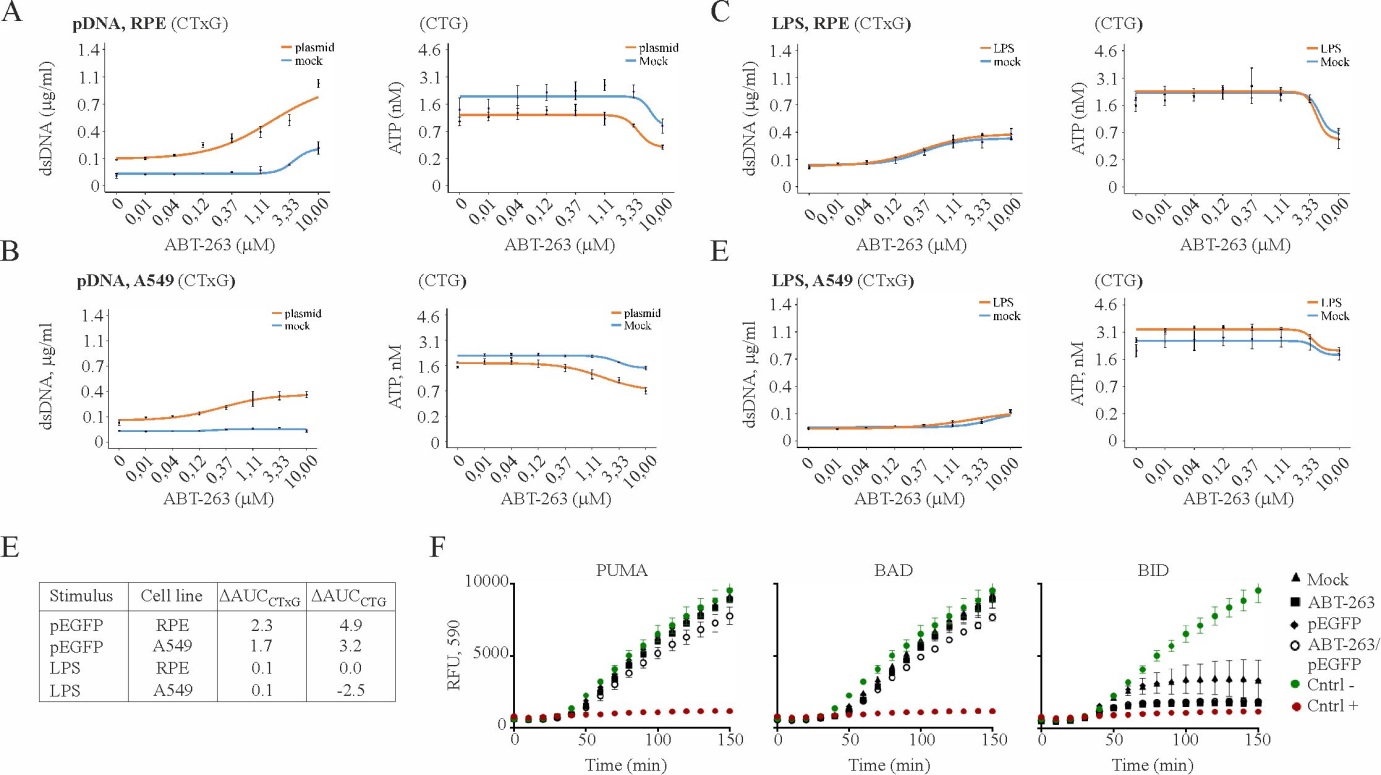


**Fig. S6**. Bcl-2i facilitate death of cells transfected with plasmid DNA but not cells treated with LPS. (A, B) CTxG and CTG plots showing the effect of ABT-263 on viability of RPE and A549 cells transfected with plasmid DNA (300 ng). Mean ± SD, n=3. (C, D) CTxG and CTG plots showing the effect of ABT-263 on viability of RPE and A549 cells stimulated with bacterial LPS (10 μg/ml). Mean ± SD, n=3. (E) Table summarizing effect of ABT-263 on viability of pDNA-transfected or LPS-stimulated RPE and A549 cells. (F) Dynamic BH3-peptide profiling showed that PUMA, BAD and BID peptides trigger MOMP in plasmid-transfected ABT-263 treated cells at 3 h post treatment/transfection, contrary to non-transfected or non-treated cells. Mean ± SD, n=3.


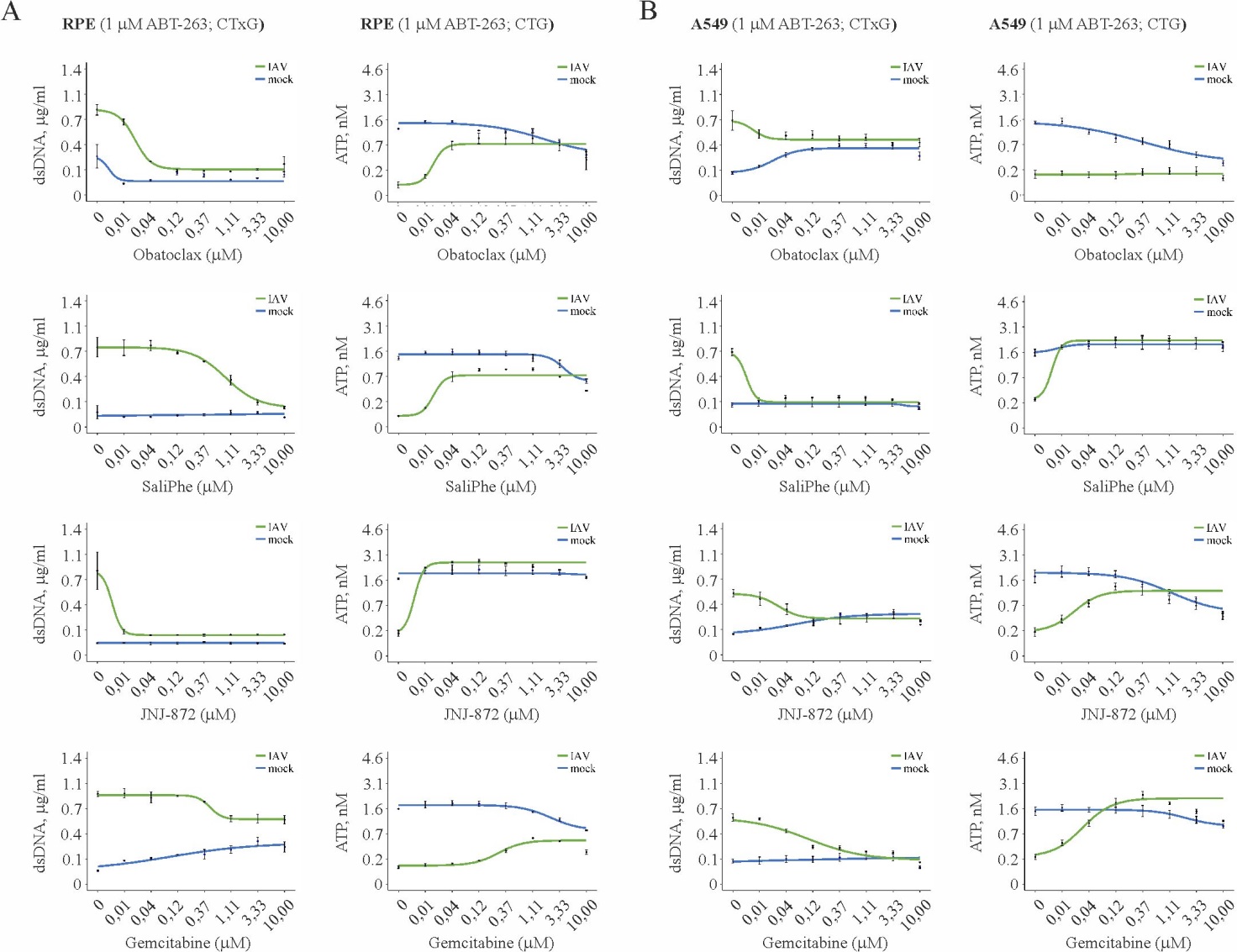


**Fig. S7**. Obatoclax, SaliPhe, JNJ-872 and gemcitabine rescued IAV-infected RPE and A549 cells from ABT-263-mediated premature death. (A, B) RPE and A549 cells were treated with ABT-263 (1 μM) and with increasing concentrations of obatoclax, SaliPhe, JNJ-872 or gemcitabine. Cells were mock- or IAV-infected (moi 3), and cell death and viability was measured using CTxG and CTG assays after 24 h post infection. Mean ± SD, n=3.

**
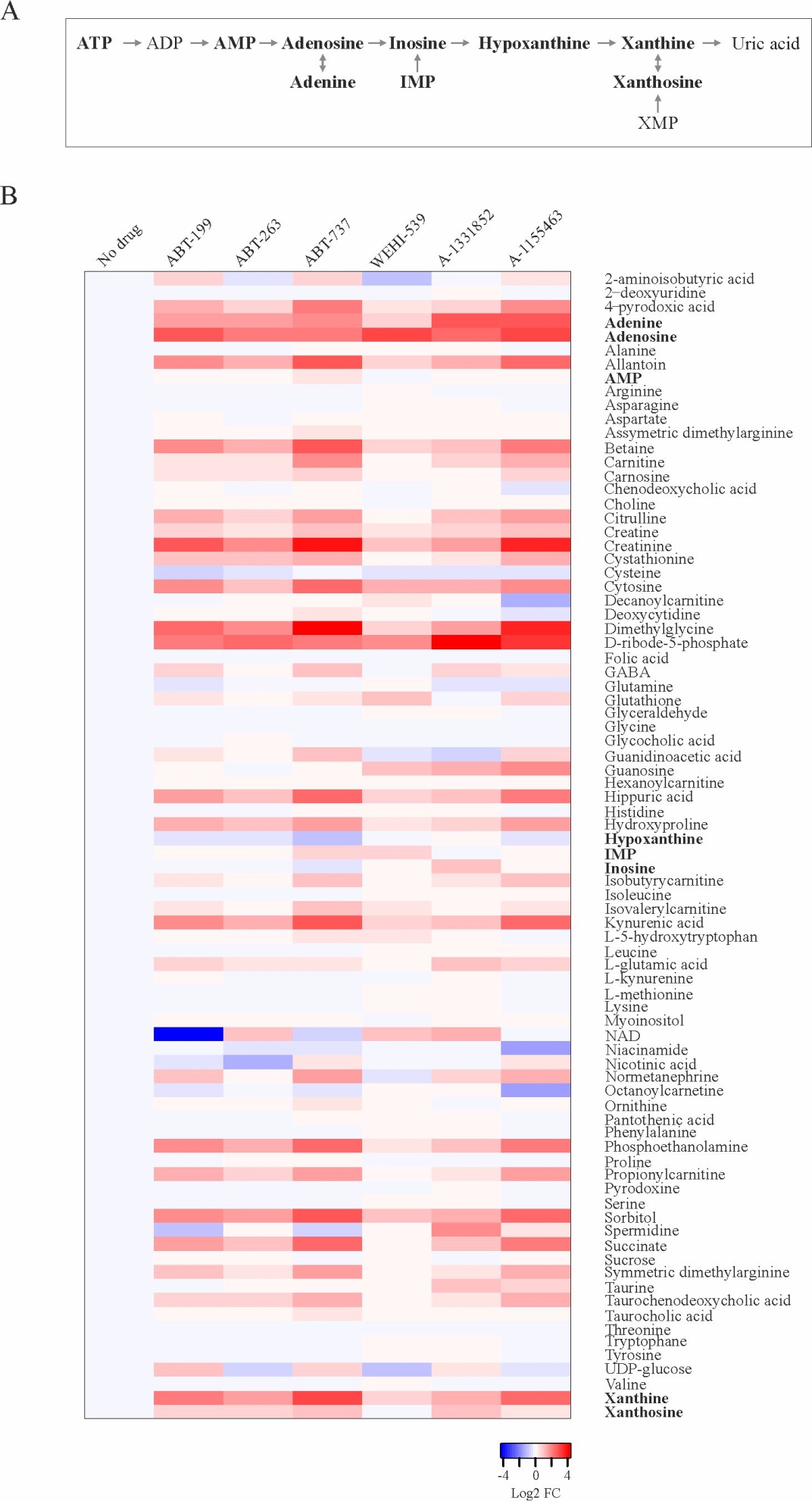
**

**Fig. S8.** Different Bcl-2i differential effect on ATP metabolism of RPE cells. (A) Simplified schematic diagram depicting the ATP metabolism. Metabolites implicated in the present study are in bold. (B) RPE cells were treated with 3 μM ABT-199, ABT-263, ABT-737, WEHI-539, A-1331852 or A-1155463 or remained non-treated. Supernatants were collected. Polar metabolites were extracted and analysed using mass spectrometry. A heat map of 87 metabolites is shown. Rows represent metabolites, columns represent samples. Each cell is colored according to the log2-transformed values of samples, expressed as FC relative to the average of mock controls. Metabolites of purine and pyrimidine metabolism are highlighted.


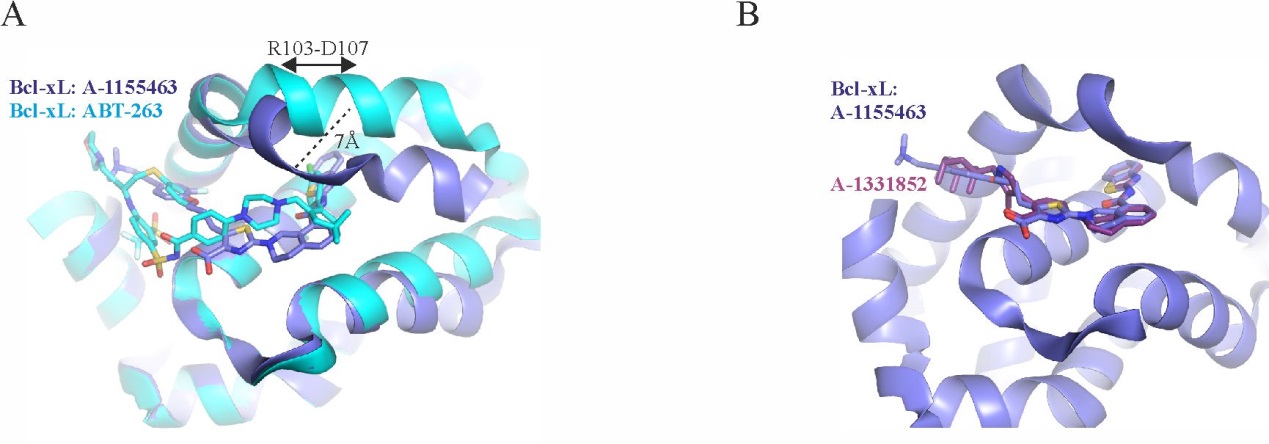


**Fig. S9**. Structures of Bcl-xL with Bcl-2i. (A) A-115463 (blue, stick) binding induces major conformational change (7 Å backbone movement) in the Bcl-xL (blue, cartoon, PDB: 4QVX) that disrupts the helical structure of the Bcl-xL in the region of R103-D107. Where is ABT-263 (light blue, stick) binding to Bcl-xl (light blue, cartoon, PDB: 4QNQ) does not induce conformational change in the helix that is seen with A-115463. (B) Predicted binding of A-1331852 (purple, stick) seems to be similar to A-115463 binding (blue, PDB 4QVX) as both compounds have benzothiazole and dihydroisoquinolin moieties.
