# Supplementary material for: Antiviral Properties of Chemical Inhibitors of Cellular Anti-Apoptotic Bcl-2 Proteins
Source: Viruses. 2017 Sep 25;9(10):271. doi: 10.3390/v9100271 (PMC5691623; doi:10.3390/v9100271)
Supplement: Supplementary file 1 [file viruses-09-00271-s001.zip › Legends for supplementary videos.docx]

**Legends for supplementary videos for** *“*Antiviral potential of chemical inhibitors of cellular Bcl-2 proteins*” by Bulanova et al.*

**Supplementary video 1**. Tracking infection (for 96 h) of pancreatic cancer cells with SFV vector VA7 virus (moi 0.01) expressing a fluorescent reporter (mCherry) in the absence of ABT-263. Green = cell death (CTxG), Red = SFV (VA7-mCherry).

**Supplementary video 2**. Tracking infection (for 96 h) of pancreatic cancer cells with SFV vector VA7 virus (moi 0.01) expressing a fluorescent reporter (mCherry) in the presence of 1 μM ABT-263. Green = cell death (CTxG), Red = SFV (VA7-mCherry).
